# 

**Published:** 1866-11

**Authors:** 


### Books and Pamphlets Received.

A Manual of Medical Jurisprudence. By Alfred Swaine Taylor, M. D., F. R. S. Sixth American from the eighth and revised London edition, with notes and references to American decisions, by Clement B. Penrose. Philadelphia: Henry C. Lea, 1866.

The Science and Practice of Medicine. By William Aitken, M. D., Edinburgh, in two volumes—vol. 1. From the fourth London edition, with additions, by Meredith Clymer, M. D., late Professor of the Institutes and Practice of Medicine in the University of New York; formerly Consulting Physician to the Philadelphia Hospital, etc. Philadelphia: Lindsay & Blakiston, 1866.

Clinical Observations on Functional Nervous Disorders. By C. Handfield Jones, M. B. Cantab.; F. R. C. P. Lond.; Physician to St. Mary's Hospital. Philadelphia: Henry C. Lea, 1867.

An Introduction to Practical Chemistry, including analysis. By John E. Bowman, F. C. S., late Professor of Practical Chemistry in King's Collefle, London. Edited by Charles L. Bloxam, F. C. S., Professor of Practical Chemistry in King's College, London, etc., etc., with one hundred and seven illustrations. Fourth American, from the fifth revised London edition. Philadelphia: Henry C. Lea, 1866.

Notes on Epidemics, for the use of the Public. By Francis Edmund Anstie, M.D., F. R. C. P., Senior Assistant Physician to the Westminster Hospital. First American edition. Philadelphia: J. B. Lippincott & Co., 1866.

Diagnosis and Prescription Record. Fourth edition. New York: W. Wood & Co., 61 Walker street, and Schiefflen & Co., 170 William street.

A Treatise on Vesico-Vaginal Fistula. By M. Schuppert, M. D., Surgeon of the Orthopædic Institute, New Orleans, La.

Inguinal Aneurism—Successful Ligation of External Iliac Artery by means of Silver Wire. By C. H. Mastin, M. D., of Mobile, Ala.

Neuralgia of the Face—Resection of the second branch of the fifth pair of nerves in the Spheno-Maxillary Fossa at the Foramen Rotundum of the Sphenoid Bone. By M. Schuppert, M. D., Surgeon of the Orthopædic Institute, New Orleans.

Transactions of the Medical Society of the State of Pennsylvania, at its Seventeenth Annual Session, held at Wilkesbarre, June, 1866.

Berkshire (Mass.) Medical College. Forty-fourth Annual Catalogue, 1866.

Prospectus of the Second Annual Course of Instruction in the St. Louis College of Pharmacy—Session of 1866–7.

On Provision for the Insane Poor of the State of New York, and the adaptation of the "Asylum and Cottage Plan" to their wants, as illustrated by the history of the Colony of Fitz James, at Clermont, France. By Charles A. Lee, M. D.

Remarks on the Bible. By John O'Reilly, M. D.

Dr. Charles W. Betzel's Circular of Truss, single and double. Patented May 9th, 1865, for the term of seventeen years. Sanctioned by Prof. S. D. Gross, in his "System of Surgery." Recommended by Surgeon General Barnes, U. S. A., by whose authority it was practically proved in one of the U. S. General Hospitals of Philadelphia. B. A. Fahnestock's Son & Co., Pittsburgh, Pa.

Revised List of the Publications of J. B. Lippincott & Co., Philadelphia.

### Exchange Journals.

The following Journals are regularly received in exchange:

London Lancet—Editors, J. H. Bennett, M. D., T. Wakely, jr., M. R. C. S. E.

Braithwaite's Retrospect of Practical Medicine and Surgery. New York: W. A. Townsend, 434 Broome street.

The Ophthalmic Review, edited by J. Z. Lawrence and Thomas Winslow, London.

American Journal of the Medical Sciences, edited by Isaac Hays, M. D.

Boston Medical and Surgical Journal, edited by Samuel L. Abbott, M. D. and James C. White, M. D.

The American Journal of Insanity, edited by the officers of the New York State Lunatic Asylum.

The New York Medical Journal.

The Cincinnati Lancet and Observer, edited by Edward B. Stevens, M. D. and John A. Murphy, M. D.

The St. Louis Medical and Surgical Journal, edited by M. L. Linton, M. D. and Frank W. White, M. D.

The Medical Record.

The Chicago Medical Journal.

The Chicago Medical Examiner, edited by N. S. Davis, M. D..

The Medical and Surgical Reporter, edited by S. W. Butler, M. D.

The Cincinnati Journal of Medicine, edited by George C. Blackman, M. D., Theophilus Parvin, M. D. and Roberts Bartholow, M. D.

The Richmond Medical Journal, edited by E. S. Gaillard, M. D. and W. S. McChesney, M. D.

The Savannah Journal of Medicine, edited by Juria Harris, M. D., J. B. Read, M. D. and J. G. Thomas, M. D.

The Medical Reporter, edited by J. S. B. Alleyne, M. D. and O. F. Potter, M. D.

The Detreit Review of Medicine and Pharmacy, edited by George T. Andrews, M. D., S. T. Duffield, M. D. and E. W. Jenks, M. D.

Atlanta Medical and Surgical Journal, edited by J. G. Westmoreland, M. D.

The Medical and Surgical Monthly, Memphis, Tenn., edited by Frank A. Ramsay, M. D., D. D. Saunders, M. D., E. Mills Willett, M. D. and William H. White, M. D.

Southern Journal of Medical Sciences, edited by E. D. Turner, M. D., D. Warren Brickell, M. D. and C. Beard, M. D.

The Pacific Medical and Surgical Journal and Press, edited by Henry Gibbons, M. D.

The Galveston Medical Journal, edited by Greenvill Dowell, M. D.

The New Orleans Medical Record, a semi-monthly Journal of the Medical Sciences, edited by Bennett Dowler, M. D. and S. R. Chambers, M, D.

The American Journal of Pharmacy, edited by William Proctor, jr.

Medical News and Library, by Henry C. Lea, Philadelphia.

The Druggist's Circular and Chemical Gazette; The Journal of Materia Medica, by Joseph Bates, M. D. and H. A. Tilden.

The Dental Cosmos, edited by J. H. McQuillen, D. D. S. and George J. Zeigler, M. D.

The Atlantic Monthly, published by Ticknor, Fields & Co., Boston.

Godey's Lady's Book, published by Louis A. Godey, Philadelphia.

The Herald of Health.

American Eclectic Medical Review, New York.

Eclectic Medical Journal, Cincinnati, Ohio.

Dental Register, Cincinnati.

The Nation.

Every Saturday, published by Ticknor, Fields & Co., Boston.

Educational Monthly.

---

Transactions of the Ohio State Medical Society, 1866.

The medical papers are reports on Military Surgery by Dr. Gay of Columbus, on Obstetrics by Dr. Reamy of Zanesville, who covers a great amount of ground and presents a very interesting and important report; on Therapeutics of Zimotic Diseases by Edward B. Stevens, editor of the *Cincinnati Lancet and Observer*, who never fails to present something valuable before the State Society. The paper this year is very suggestive and instruetive. There are reports upon Incurable Diseases, Public School Instruction, and some reports of surgical cases. all very creditable to the Ohio State Medical Society as well as to their respective authors.

